# Anaemia Prevalence More Than Doubles in an Academic Year in a Cohort of Tertiary Students: A Repeated-Measure Study in Cape Coast, Ghana

**DOI:** 10.1155/2022/4005208

**Published:** 2022-01-22

**Authors:** Regina Elorm Amoaning, Ernestina Siaw Amoako, Grace Arezie Kyiire, Dennis Dela Owusu, Happy Bruce, David Larbi Simpong, Patrick Adu

**Affiliations:** Department of Medical Laboratory Science, School of Allied Health Science, University of Cape Coast, Cape Coast, Ghana

## Abstract

**Background:**

The stress of academic life may predispose young adults to poor dietary habits, which could potentially precipitate nutritional deficiencies, such as iron deficiency. This study evaluated factors predictive of optimal iron stores as well as changes in haematological parameters over the course of an academic year in a cohort of tertiary students.

**Materials and Methods:**

The repeated-measure cohort study recruited 117 undergraduate students from September 2018 to May 2019. Venous blood samples were drawn for full blood count estimation, qualitative glucose-6-phosphate dehydrogenase (G6PD) status, haemoglobin variants, and blood group determination during the first 2 weeks of semester 1. However, anthropometric parameters as well as full blood counts were determined for each participant during the first week and last week of semesters 1 and 2. Additionally, semistructured questionnaires were used to capture sociodemographic data. Also, serum ferritin was estimated for each participant using enzyme-linked immunosorbent assay.

**Results:**

Overall, 23.1% and 15.5% of participants inherited G6PD defect (G6PDd) or haemoglobin variants, respectively. However, group O (68/117; 58.1%) was the predominant ABO blood group and an overwhelming 90.6% (106/117) inherited Rh D antigen. The prevalence of anaemia increased from 20% at the beginning of the first semester to 45.1% at the latter part of the second semester. G6PDd participants had significantly higher median serum ferritin than G6PD normal participants (*p* = 0.003). Also, a significantly higher proportion of females were iron depleted (25% vs. 2.3%) or iron deficient (14.3% vs. 9.3%) compared to males. Moreover, being male, G6PD deficient, or 21–25 years was associated with increased odds of participants having optimal serum ferritin levels.

**Conclusion:**

The progression of anaemia prevalence from mild to severe public health problem over the course of one academic year should urgently be addressed.

## 1. Introduction

Adequate nutrition is very important in physical and mental development of humans. Poor nutrition has been shown to lead to reduced productivity and increased susceptibility to disease as a consequence of reduced immunity, as well as impaired physical and mental development [1]. The nutritional status of individuals is greatly influenced by dietary intake as well as physical activity. Young adults may be particularly affected by poor dietary choices due to spouts of physical growth and the development of higher cognitive functions, such as abstraction and criticality [1, 2]. Previous studies have demonstrated that the stress of academic activities predisposes university students to adoption of unhealthy eating habits, such as meal skipping, snacking, and frequent fast-food consumption [3]. Furthermore, other studies have even found associations between unhealthy dietary intake and poor academic performance as well as cardiovascular diseases [4–6].

Poor dietary habits may lead to micronutrient deficiencies, such as iron deficiency. Generally, iron deficiency is a prevalent condition among the populace in sub-Saharan Africa. Poor nutritional choices in tertiary students may thus exacerbate this public health problem over the course of the stress-laden academic year. As iron is required for a host of biochemical processes including haemoglobin formation, iron deficiency may lead to anaemia with a consequent impaired cognitive function. In spite of the obvious fact that academic stress could potentially lead to nutritional imbalances in tertiary students, there is scarcity of studies addressing the nutritional challenges faced by these young adults. This study aimed to track the changes in anthropological and haematological parameters as well as determine factors predictive of optimal iron stores in a cohort of university students over the course of one academic year. We sought to provide empirical data that will educate students on the need for consistent healthy eating habits as well as inform the need or otherwise for specific intervention. We employed a repeated-measure cohort design to mitigate the inherent major weakness of previous cross-sectional studies that only provided snapshots of the dynamics in study variables.

## 2. Materials and Methods

### 2.1. Study Design

This was a repeated-measure prospective cohort study that was conducted from September, 2018, to May, 2019. The study was undertaken at the University of Cape Coast, in the Department of Medical Laboratory Science (MLS). The MLS Department has a total student population of 376. Overall, 117 Medical Laboratory Science students (second to fourth year) of the University of Cape Coast were conveniently recruited into the study. Participants were sampled during the second and last weeks of both semester one and semester two; in all, a total of 71 participants completed all the four sampling.

### 2.2. Exclusion Criteria

The study excluded students with clinically diagnosed malabsorption syndromes, liver diseases, renal diseases, those with viral diseases, and those with subclinical inflammation as indicated by C-reactive protein measurement. Students who showed signs and symptoms of illness such as weakness, shivering, cough, and other symptoms were also excluded. Additionally, all level 100 students (78 of 376 students) were excluded from the study as they were considered to be in the process of adapting to their new environment.

### 2.3. Demographic and Dietary Data Collection

A semistructured questionnaire was used to take the demographic and lifestyle information of the participants.

### 2.4. Anthropometric Measurement

The body mass index (BMI) of participants was determined using basic parameters, namely, height and weight. An Omron health scale (OMRON, Japan) and a stadiometer were used to measure the weight (to the nearest 0.1 kg) and height (to the nearest 0.1 m), respectively. The BMI was then calculated using the following formula:

(1) $$\begin{array}{c} \text{BMI }\left(\text{kg}/{\text{m}}^{2}\right)=\frac{\text{weight}\left(\text{kg}\right)}{\text{height}\left(\text{m}\right)\times \text{height}\left(\text{m}\right)}. \end{array}$$

Body mass indices obtained were subsequently classified in accordance with WHO standard of classification of BMI.

### 2.5. Blood Sample Collection and Laboratory Analysis

Five (5) ml of whole blood was collected from each participant by venepuncture following standard phlebotomy protocols. About 1.5 ml of the sample collected was dispensed into SST II clot activator tubes and the remaining 3.5 ml was dispensed into EDTA anticoagulated tube. The sample in the EDTA tube was used for the analysis of full blood count, qualitative glucose-6-phosphate dehydrogenase assay, sickling slide test, haemoglobin electrophoresis assay, and blood grouping. The sample in the SST II serum separator tube was used for the analysis of ferritin and C-reactive protein levels.

### 2.6. Glucose-6-Phosphatase Deficiency (G6PD) Test

The qualitative G6PD status screening was undertaken using the methaemoglobin reductase assay in accordance with published protocols [7]. As a quality control, positive and negative controls were run for each participant.

### 2.7. Sickling Slide Test

The sickling status of the participants was assessed using the sodium metabisulfite method according to published protocols [7].

### 2.8. Haemoglobin Electrophoresis

The haemoglobin type of participants was determined using the cellulose acetate electrophoresis procedure as previously described [7]. For each electrophoretic run, control samples composed of haemoglobin A, C, S, and F were run to enable haemoglobin identification.

### 2.9. Blood Grouping (Tile Forward Typing Method)

The ABO and Rh D blood group of each participant was determined using Accucare Monoclonal Antibodies (Labcare Diagnostics, India) via the tile method in accordance with the manufacturer's protocol.

### 2.10. Full Blood Count (FBC)

The full blood count and differential cell counts of the participants were estimated using Mindray BC-2800 (Mindray, China) according to the manufacturer's instructions. Gender-specific reference ranges were used to classify the haemoglobin concentration of participants; females and males were, respectively, classified as anaemia per haemoglobin cutoff of <11.5 g/dL and <12.5 g/dL.

### 2.11. Ferritin Analysis

The human ferritin enzyme immunoassay test kit (Chemux Bioscience, Inc., USA) was used to analyse the ferritin levels of each participant. The test was conducted according to the manufacturer's instructions and the absorbance was read with the Multiskan EX Primary EIA microplate reader version 2.3 (Thermo Fisher Scientific Inc., USA). A standard curve drawn from the results of the absorbances of manufacturer-specific standards was used to extrapolate the respective ferritin concentrations of participants.

### 2.12. C-Reactive Protein Measurement

Serum C-reactive protein (CRP) was estimated using the ELISA method in accordance with the manufacturer's specifications (R&D Systems China Co., Ltd., China). Plates were read on the URIT-660 Microplate Reader (URIT Medical Electronic Co., Ltd., Guangxi, China). As per WHO recommendation, CRP cutoff for no inflammation was taken as ≤5 mg/l [8].

### 2.13. Data Analysis

The data collected from the anthropometric measurement and answers to the questionnaire together with the laboratory assays were analysed using the SPSS version 25.0 (IBM Corp., USA) and GraphPad Prism 6.01 (GraphPad software Inc., USA). The normality of the data obtained was tested using D'Agostino and Pearson's omnibus normality test. Descriptive statistics, correlational analyses, and Chi-square test were used to explore the data. Also, binary logistic regression analyses were used to estimate the factors associated with optimal serum ferritin levels (ferritin >30 ng/ml). Serum ferritin levels were compared using the Mann–Whitney test (for two group comparison) or Kruskal–Wallis with Dunn's multiple comparison test (for >2 group comparison). Statistical significance was established at *p* < 0.05 at 95% confidence interval using the two-tailed assumptions.

## 3. Results

Table 1 describes the general characteristics of the participants of this study. The majority of the participants (81/117; 69.2%) were between the ages of 21 and 25 years with a greater percentage (71/117; 60.7%) being males. Also, whereas 23.1% of participants had qualitative glucose-6-phosphate dehydrogenase defect, 15.5% (13/117) inherited haemoglobin variants. The most dominant ABO blood type was blood group O (68/117; 58.1%), whereas 90.6% (106/117) inherited the Rh D antigen.

**Figure 1:**
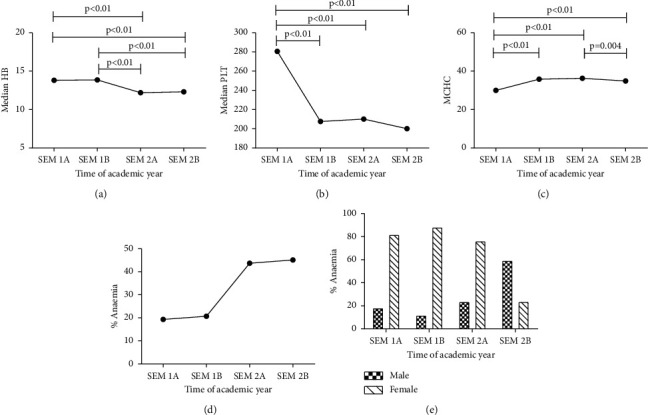
The trend of the measured parameters during one academic year. (a) The trend of haemoglobin concentration. (b) The trend of the change in platelet count. (c) The pattern of the change in MCHC of participants. (d) The prevalence of anaemia among the participants of the study at each time point. (e) The gender-wise distribution of anaemia prevalence (Semester 1A, early part of first semester; Semester 1B, latter part of first semester; Semester 2A, early part of second semester; Semester 2B, latter part of second semester (Kruskal–Wallis with Dunn's multiple comparison test was performed)). HB: haemoglobin, MCHC: mean cell haemoglobin concentration; PLT: platelet, and BMI: body mass index.

Figure 1 illustrates the trend of the various parameters measured during the academic year. Although the median haemoglobin levels did not significantly change within the same semester, the median haemoglobin levels significantly dropped between the first and the second semester (Figure 1(a)). Also, the platelet count of the participants significantly reduced (*p* < 0.01) at the end of the first semester (Figure 1(b)); however, there were no significant changes in platelet count between the latter part of semester one and throughout semester two within the academic year. Additionally, the MCHC of the participants significantly increased during semester one (*p* < 0.001; Figure 1(c)) and remained high but significantly reduced during the latter part of semester two (*p* = 0.004). The prevalence of anaemia increased from 20% at the beginning of semester one and reached the highest value of 45.1% at the latter part of the second semester (Figure 1(d)). Moreover, anaemia was more prevalent in females at all the time points, except at the latter part of semester two where a higher proportion of males were anaemic (Figure 1(e)).

**Figure 2:**
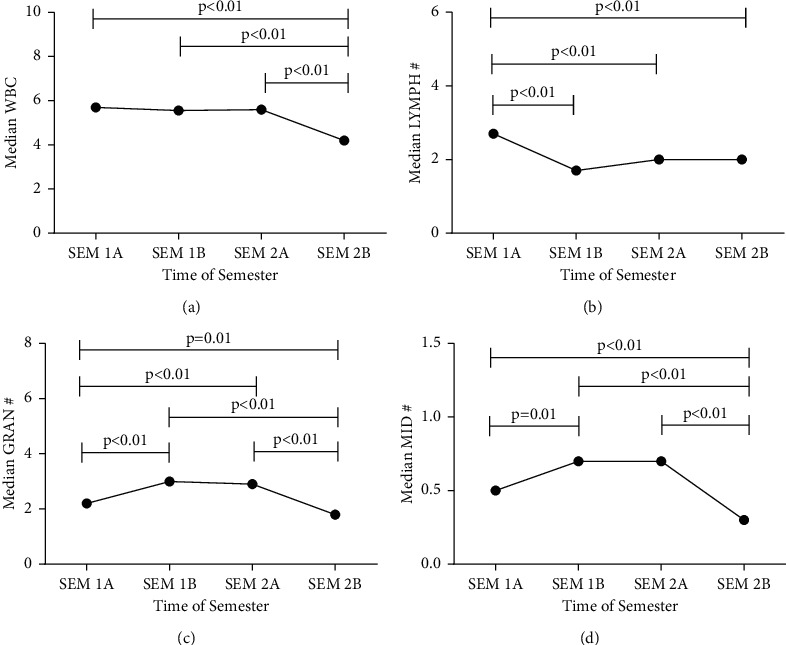
The trend of the white blood cell differentials during one academic year. (a) The trend of total WBC count. (b) The trend of the change in absolute lymphocyte count. (c) The pattern of the change in absolute granulocyte count of participants. (d) The pattern of the change in the absolute MID count of participants. Statistical comparisons were undertaken using Kruskal–Wallis with Dunn's multiple comparison test (Semester 1A, early part of first semester; Semester 1B, latter part of first semester; Semester 2A, early part of first semester; Semester 2B, latter part of second semester; WBC, white blood cell; #, absolute; MID: a collective term for monocytes, basophil, and eosinophil cells).

Figure 2 shows the trend of white blood cell differential parameters during the study period. Generally, there was no significant change in the median total WBC count until the latter part of semester two in which the total WBC count significantly decreased (Figure 2(a)). However, whereas the absolute lymphocyte counts significantly decreased during semester one (Figure 2(b)), the absolute lymphocyte counts marginally increased during semester two. Moreover, the counts of both granulocytes (Figure 2(c)) and MID cells (Figure 2(d)) cells significantly increased during the latter part of semester one and then significantly reduced during the latter part of semester two.

**Figure 3:**
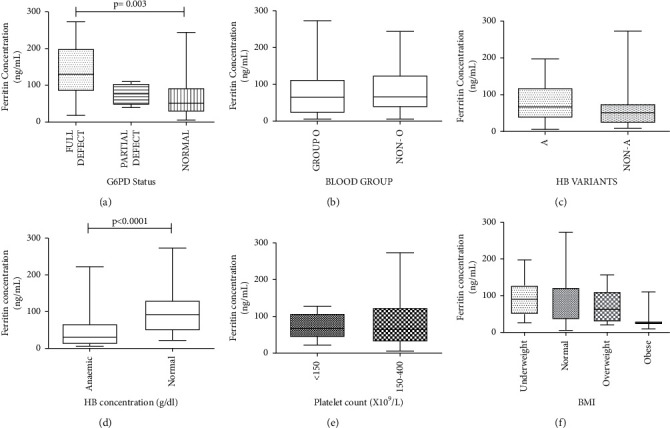
Serum ferritin levels of participants stratified according to BMI and haematological parameters. (a) The distribution of the ferritin levels of participants across their G6PD status (Kruskal–Wallis with Dunn's multiple comparison test was performed). (b) The distribution of the ferritin levels of participants across their blood groups. (c) The distribution of the ferritin levels of participants across their haemoglobin types. (d) The distribution of the haemoglobin concentration of the participants across the different groups of ferritin concentration (the Mann–Whitney test was used). (e) The distribution of the platelet count of the participants across the different groups of ferritin concentration. (f) The distribution of the body mass index of the participants across the different groups of ferritin concentration. BMI: body mass index; PLT: platelet; and HB: haemoglobin.

Figure 3 illustrates the serum ferritin levels of participants stratified per the various estimated participant parameters. The median ferritin concentration of participants with defective glucose-6-phosphate dehydrogenase (G6PD) status was significantly higher than the median ferritin level of participants with normal G6PD status (*p* = 0.003; Figure 3(a)). However, the median ferritin levels of participants did not significantly differ when participants were stratified based on ABO blood group (Figure 3(b)) or inherited haemoglobin variants (Figure 3(c)). The median ferritin levels of participants were significantly lower (Figure 3(d)) in participants with anaemia. However, the median ferritin levels did not significantly change when participants were stratified based on platelet counts (Figure 3(e)) or BMI (Figure 3(f)) categorization.

The data was further explored per participant serum ferritin levels (Table 2), namely, iron depletion (serum ferritin <15 ng/ml), iron deficiency (serum ferritin ≥15–<30 ng/ml), or normal (serum ferritin ≥30 ng/ml). A significantly higher proportion of females were iron depleted (25% vs. 2.3%) or iron deficient (14.3% vs. 9.3%) than males. Also, whereas 30.8% and 19.5% of anaemic participants had iron depletion and iron deficiency, respectively, only 6.7% of participants with normal haemoglobin levels were iron deficient. Moreover, a significantly higher proportion of non-O blood-type individuals were iron depleted compared to individuals of blood group O (*p* = 0.005; 16.3% vs. 0%). Additionally, whereas only 5% of Rh D positive individuals were iron depleted, 45.5% of Rh D negative individuals were iron depleted. Furthermore, whereas 13.5% participants with qualitative G6PD normal function had iron depletion, none of the G6PD-deficient individuals had depleted iron stores.

We observed a positive correlation between serum ferritin and all the FBC parameters except platelet count (*r* = −0.164). However, only the correlation between haematocrit (HCT, *p* = 0.01), mean cell haemoglobin (MCH, *p* = 0.01), absolute MID cell count (MID, *p* = 0.01), and ferritin were statistically significant (see Supplementary Table 1). To further understand what factors might be predictive of optimal serum ferritin levels in the participants, the data was further explored using binary logistic regression analyses (Table 3). The analyses showed that being G6PDd (OR: 54.334, *p* = 0.066), non-O blood group (OR: 24.843, *p* = 0.091), male (OR: 56.726; *p* = 0.039), or between 21 and 25 years (OR: 9.891; *p* = 0.324) was associated with increased odds of participant having optimal serum ferritin levels. However, having a haemoglobin variant (OR: 0.098; *p* = 0.129), anaemia (OR: 0.243; *p* = 0.296), or thrombocytopenic (OR:0.593; *p* = 0.787) was associated with reduced odds of a participant having optimal serum ferritin levels.

## 4. Discussion

The demands of academic activities may predispose young adults to poor dietary habits which could potentially precipitate nutritional deficiencies, such as iron deficiency (Jahan et al., 2018). In spite of the obvious fact that academic stress could potentially lead to nutritional imbalances in tertiary students, there is scarcity of studies addressing the nutritional challenges faced by these young adults. Using a cohort of university students, we showed that the activities in an academic year were associated with significant alterations in the haematological and biochemical profiles of these students. Overall, our study showed that whereas anaemia prevalence increased over the course of the academic year, 22.5% (16/71) of the students had suboptimal iron stores at the latter part of the academic year, with the majority being disproportionately females.

The incidence of anaemia more than doubled over the course of a single academic year, increasing from 20% at the beginning of the academic year to 45% at the latter part of the academic year. When we compared our data to the WHO categorization of public relevance of anaemia, there was a transition from mild (20% prevalence) to a severe public health problem [9] in a single academic year. The trend of anaemia reported in the present study is indicative of a worsening nutritional status over the course of the academic year. Not surprisingly, and in line with the generally higher incidence of anaemia in females compared to males, females generally constituted a higher proportion of the anaemic individuals. It is noteworthy that the majority of the students indicated preparing their own meals while on campus. Even though this might provide a sure way of ensuring food quality, it is equally valid to argue that either the nutritional content of the self-made meals generally declined or that the students became negligent during the course of the semester as academic work intensified. Although the study did not employ food frequency questionnaire to quantify the nutritional value of food consumed whilst students were on campus, we are of the view that stress due to academic activities predisposes students to abandon healthy dietary needs. Our finding that 30.8% and 19.2% of the students, respectively, had iron-depleted erythropoiesis and iron-deficiency anaemia at the latter part of the academic year provides additional evidence to support our claim of poor dietary intake among these students during the academic year. Whereas only 7.3% of the participants ate breakfast regularly whilst on campus, an overwhelming majority (92.7%) indicated either always or sometimes skipping breakfast during the academic year (see Supplementary Table 2). Even though the students recruited for this study represent only a fraction of the entire student population, we believe that the overall picture in the general student population may not be different. Since academic excellence could be impacted by nutritional deficiency, our study highlights a public health challenge that needs addressing in these young adults. We propose that a larger, longitudinal cohort study that systematically evaluates nutritional status of these tertiary students at regular intervals would enable a better understanding of the nutritional challenges to inform policy decisions. Such a study should not be restricted to tertiary levels but must as well include second cycle institutions in its sampling frame as nutritional deficiencies in the teenage might have lifelong impact.

In agreement with a previous study undertaken in the subregion [10], we found 23.1% of the participants being G6PD deficient. Noteworthily, participants with defective G6PD deficiency generally had higher serum ferritin levels compared to G6PD normal individual. Furthermore, none of the participants with depleted iron stores had inherited G6PD enzymopathy. This finding of higher serum ferritin levels in G6PD-deficient individuals has previously been reported in other settings [11, 12]. Our logistic regression analyses provided further evidence to show that G6PD deficiency was associated with greater odds of participant having optimal iron stores (serum ferritin ≥30 ng/ml). This is not surprising considering that G6PD deficiency only leads to intravascular haemolysis in the presence of oxidant stressors in which released iron is generally recycled. As G6PD deficiency is prevalent in the study area, we speculate that this selectively high median serum ferritin in G6PD-deficient individuals might have been part of the traits evolved to offer survival advantages against endemic diseases, like malaria [13, 14]. However, a larger cohort study will have to be undertaken to investigate the validity and the generalizability of this assertion.

It is interesting to note that although participants who were Rh D-negative phenotype constituted only 9.4% of the study population, these individuals constituted a higher proportion of the participants with depleted iron stores. Although our sample size in terms of the proportion of participants with Rh D-negative phenotype does not allow for drawing categorical inferences, it will be interesting to explore this finding in future well-controlled studies. Additionally, our study found evidence that the proportion of participants with a non-O-type blood group with depleted iron stores were significantly higher than blood group O type individuals. This is in contrast with a previous study in Enugu State, Nigeria, which found that serum ferritin levels were significantly reduced in blood group B individuals compared to group O or A individuals [15]. We believe this is an area that warrants further exploration to inform patient public health decision making processes as studies investigating the impact of blood group on iron stores are scanty. Previous studies have found lower incidence of anaemia in blood group O individuals compared to other ABO blood type [16]. In a study that assessed incidence of anaemia in blood donors in Cameron, Kwenti and Kwenti also reported blood group O to be the least represented, with blood group AB being the predominant group [17]. However, in a previous study among a cohort of students in Bidar, India, Reshmarani and associates rather found blood group A to be the least represented in anaemic individuals [18]. Although these studies highlight a potential influence of blood type on incidence of anaemia, more studies are needed to establish causal relationships.

Our study also found alterations in leucocyte and platelet counts during the academic year. Many studies have previously found associations between stress and specific alterations in full blood count parameters. For example, stressful events have been shown to lead to an increase in circulating catecholamines that increases platelet activation resulting in decreasing platelet counts through the activation of *α*-2 adrenoreceptors [19, 20]. Also, people with generalized anxiety disorders, which are associated with a lot of mental stress, showed low platelet counts and high mean platelet volume [19]. A review written by Koudouovoh-Tripp and Sperner-Unterweger also reported increased platelet activity during mental stress [21]. However, in relation to white blood cell counts, a previous study found an inverse relationship with the hours of work [22]. Another study conducted by Naoko and Hisataka rather showed a significant positive correlation between psychological stress and WBC or neutrophil counts [23]. Although the study reported herein did not estimate the psychological stress in our cohorts, we found that the total white blood cell counts of the participants significantly decreased by the end of the academic year. This was also associated with significant changes in differential leucocyte counts over the academic year. It must be pointed out that 74% of the participants reported being stressed during the course of the academic year (Supplementary Table 3). The significance and causal associations of the alterations in total leucocyte count as well as differential leucocyte counts remain to be explored in future studies.

Our inability to include food frequency questionnaires made it difficult to track the trend in the eating habits of the students. For example, male participants had low anaemia prevalence throughout most part of the academic year, except at the latter part of semester 2, where this trend was reversed Food frequency questionnaires (FFQ) evaluation might have provided plausible reasons for the observed trend; however, FFQ administration was not part of the present study design. Also, our findings are clearly indicative of alterations of full blood count parameters during the course of the academic year. For example, platelet counts, lymphocyte counts, and total white blood cell counts decrease over the course of the academic year. However, in view of the observational nature of the study reported herein, we could not assign causality to our findings. Furthermore, the small sample size (due to about 36% dropout of participants) and the fact that this is a single-centre study limit the generalizability of the findings presented herein. In spite of these acknowledged limitations, our study highlights that anaemia prevalence progressively worsens from mild to severe public health problem over the course of an academic year. Our findings are suggestive of a need for a systematic public health campaign to educate tertiary students about the relationship between healthy nutrition, better cognitive function, and improved academic performance. Moreover, this baseline data could inform future, well-controlled causality studies to inform policy formulation in the study area.

## Figures and Tables

**Table 1:** The demographic characteristics of the participants.

Characteristic		*N* (%)
Age (years)	16–20	30 (25.6)
	21–25	81 (69.2)
	26–30	4 (3.4)
	31–35	2 (1.7)

Sex	Female	46 (39.3)
	Male	71 (60.7)

Marital status	Married	4 (3.4)
	Single	113 (96.6)

Qualitative G6PD status	Normal	90 (76.9)
	Partial defect	4 (3.4)
	Full defect	23 (19.7)

Haemoglobin types	A	71 (84.5)
	AC	3 (3.6)
	AS	8 (9.5)
	^*∗*^AS/AD	1 (1.2)
	SC	1 (1.2)

ABO blood type	A	25 (21.4)
	B	20 (17.1)
	AB	4 (3.4)
	O	68 (58.1)

Rh D type	D positive	106 (90.6)
	D negative	11 (9.4)

G6PD: glucose-6-phosphate dehydrogenase; Rh: rhesus; and ^*∗*^one sample tested negative for sickling slide test but showed a band at the S region on cellulose acetate electrophoresis.

**Table 2:** The relationship between ferritin, gender of participants, and inherited red cell parameters.

		Ferritin			*p* value
		Iron depletion	Iron deficiency	Normal	
Gender	Female	7 (25.0)	4 (14.3)	17 (60.7)	0.007^*∗∗*^
	Male	1 (2.3)	4 (9.3)	38 (88.4)	

Level	200	1 (7.0)	2 (14.0)	11 (79.0)	0.965
	300	4 (12.0)	3 (36.0)	26 (79.0)	
	400	3 (12.5)	3 (12.5)	18 (75.0)	

Hb classification	Anaemic	8 (30.8)	5 (19.2)	13 (50.0)	**<0.001** ^*∗∗*^
	Normal	0 (0.0)	3 (6.7)	42 (93.3)	

ABO blood type	Non-O	8 (16.3)	2 (4.1)	39 (79.6)	**0.005** ^*∗∗*^
	O	0 (0.0)	6 (27.3)	16 (72.7)	

Rh D type	D positive	3 (5.0)	8 (13.3)	49 (81.7)	**0.001** ^*∗∗*^
	D negative	5 (45.5)	0 (0)	6 (54.5)	

HB type	A	5 (10.6)	8 (17.0)	34 (72.3)	0.527
	Non-A	2 (25.0)	0 (0.0)	6 (75.0)	

Qualitative G6PD status	Normal	7 (13.5)	6 (11.5)	39 (75.0)	0.449
	Defective	0 (0.0)	2 (11.1)	16 (88.9)	

^*∗∗*^Values are significant at *p* < 0.01. ^*∗*^Values are significant at *p* < 0.05. Results are reported as *n* (%). G6PD: glucose-6-phosphate dehydrogenase; HB: haemoglobin; IDA: iron depletion anaemia; and Rh D: rhesus. Groups were compared using the Chi-square test.

**Table 3:** Factors associated with optimal iron levels (ferritin ≥30 ng/mL).

	Regression coefficients	Standard error	*t*	*p* value	Odds ratio	95% confidence interval	
						Lower	Upper
Constant	−0.181	2.468	0.005	0.942	0.835		

Age							
16–20	0.613	2.563	0.057	0.811	1.846	0.012	280.347
21–25	2.292	2.324	0.972	0.324	9.891	0.104	941.532
26–30	Referent						

Gender							
Male	4.038	1.953	4.275	**0.039** ^*∗*^	56.727	1.234	2608.302
Female	Referent						

Level							
400	−3.853	2.401	2.575	0.109	0.021	0.000	2.347
300	−2.515	2.038	1.522	0.217	0.081	0.001	4.392
200	Referent						

HB							
Anaemia	−1.416	1.356	1.090	0.296	0.243	0.017	3.463
Normal	Referent						

PLT							
<150	−0.523	1.933	0.073	0.787	0.593	0.013	26.193
150–400	Referent						

Hb elect							
Non-Hb A	−2.326	1.532	2.306	0.129	0.098	0.005	1.966
Hb A	Referent						

Blood group							
Non-O	3.213	1.903	2.851	0.091	24.843	0.596	1034.693
O	Referent						

G6PD status							
Defective	3.995	2.173	3.379	0.066	54.334	0.767	3846.692
Normal	Referent						

^*∗*^Value significant at *p* < 0.05.

## Data Availability

The dataset used and/or analysed during the current study is available from the corresponding author upon reasonable request.

## Supplementary Materials

Supplementary Materials Supplementary Table 1: correlations between serum ferritin and full blood count at the end of second semester. Supplementary Table 2: dietary patterns of students. Supplementary Table 3: dietary habits of students.
