# Supplementary material for: Anaemia Prevalence More Than Doubles in an Academic Year in a Cohort of Tertiary Students: A Repeated-Measure Study in Cape Coast, Ghana
Source: Adv Hematol. 2022 Jan 22;2022:4005208. doi: 10.1155/2022/4005208 (PMC8800615; doi:10.1155/2022/4005208)
Supplement: Supplementary Materials — Supplementary Table 1: correlations between serum ferritin and full blood count at the end of second semester. Supplementary Table 2: dietary patterns of students. Supplementary Table 3: dietary habits of students. [file 4005208.f1.docx]

**Supplementary files**

Supplementary table 1 shows the relationship between serum ferritin estimated at the end of semester 2 and the corresponding FBC parameters. We observed that, there was a positive correlation between serum ferritin and all the FBC parameters except platelet count (r = -0.164). Of all these correlations, only the correlation between haematocrit (HCT, p = 0.01), mean cell haemoglobin (MCH, p = 0.01), absolute MID cell count (MID, p = 0.01) and ferritin were statistically significant.

**Supplementary table 1: Correlations between serum ferritin and full blood count at the end of second semester**

|  | | Ferritin (ng/mL) |
| --- | --- | --- |
| **WBC (10^9/L)** | r | 0.119 |
|  | p | 0.322 |
|  |  |  |
| **HGB (g/dl)** | r | 0.223 |
|  | p | 0.061 |
|  |  |  |
| **HCT (%)** | r | 1.000* |
|  | p | **0.01** |
|  |  |  |
| **MCV (fL)** | r | 0.225 |
|  | p | 0.060 |
|  |  |  |
| **MCH (pg)** | r | 0.622* |
|  | p | **0.01** |
|  |  |  |
| **PLT (X10^9)** | r | -0.164 |
|  | p | 0.173 |
|  |  |  |
| **Lymphocyte count (x10^9^/L)** | r | 0.146 |
|  | p | 0.226 |
|  |  |  |
| **MID cells (x10^9^/L)** | r | 0.336* |
|  | p | **0.01** |
|  |  |  |
| **Granulocytes count (x10^9^/L)** | r | 0.092 |
|  | p | 0.443 |
|  |  |  |

*Values are significant at p<0.05; WBC, White Blood Cell; HGB, Haemoglobin; HCT, Haematocrit; MCV, Mean Cell Volume; MCH, Mean Cell Haemoglobin; PLT, Platelet; LYMPH #, Absolute Lymphocyte Count; MID #, Absolute MID Count; GRAN #, Absolute Granulocyte Count. Parameters were compared using the Spearman’s correlation coefficient

Supplementary table 2 describes the dietary patterns of the participants. Most of the students did some sort of physical activity while 87.5% reported eating vegetables. 50% of the participants reported eating from vendors, 79.2% ate fast foods, 64.6% ate less than 3 meals daily whereas 80.2% ate snacks at least once daily.

**Supplementary Table 2: Dietary patterns of students**

| **Variable** |  | **N (%)** |
| --- | --- | --- |
|  |  |  |
| **How many times a week do you do physical activities?** | Once | 24 (25.0) |
|  | More than once | 57 (59.4) |
|  | Never | 15 (15.6) |
|  |  |  |
| **How often do you take fruits and vegetables during your stay on campus?** | Everyday | 16 (16.7) |
|  | Once a week | 66 (68.8) |
|  | 3 times a week | 1 (1.0) |
|  | Not often | 1 (1.0) |
|  | Never | 12 (12.5) |
|  |  |  |
| **What type of food do you normally eat during your stay on campus?** | Food from vendors | 3 (3.1) |
|  | Food prepared by self | 48 (50) |
|  | Both | 45 (46.9) |
|  |  |  |
| **How often do you eat fast foods (Noodles, Packed food, etc.)** | Every week | 29 (30.2) |
|  | On monthly basis | 45 (46.9) |
|  | Occasionally | 2 (2.1) |
|  | Never | 20 (20.8) |
|  |  |  |
| **How many meals apart from snacks do you eat daily?** | 1 | 5 (5.2) |
|  | 2 | 57 (59.4) |
|  | 3 | 34 (35.4) |
|  |  |  |
| **How often do you snack daily?** | Once | 56 (58.3) |
|  | More than once | 21 (21.9) |
|  | Never | 19 (19.8) |
|  |  |  |

Supplementary table 3 explores the dietary habits of students. 40.6% students skipped breakfast always with 52.1% skipping breakfast a few times. 92.7% reported eating at night with 99% reporting feeling stressed in the semester. 61.5% and 12.5% of students reported feeling stressed at the latter part of the semester and during examinations. 25% also reported feeling stressed throughout the course of the semester.

**Supplementary Table 3: Dietary habits of students**

| Variable |  | N (%) |
| --- | --- | --- |
| **How often do you skip breakfast during your stay on campus?** | Always | 39 (40.6) |
|  | Few times | 50 (52.1) |
|  | Never | 7 (7.3) |
|  |  |  |
| **Do you eat at night?** | Always | 12 (12.5) |
|  | Sometimes | 77 (80.2) |
|  | Never | 7 (7.3) |
|  |  |  |
| **Do you feel stressed during the semester?** | Yes | 71 (74.0) |
|  | No | 1 (1.0) |
|  | A few times | 24 (25.0) |
|  |  |  |
| **What time of the semester do you feel stressed?** | Early part | 0 (0.0) |
|  | Latter part | 59 (61.5) |
|  | During examinations | 12 (12.5) |
|  | Throughout the semester | 24 (25.0) |
|  | Not applicable | 1 (1.0) |
|  |  |  |
